# Functional and Cognitive Improvement After an Intensive Inpatient Multidisciplinary Rehabilitation Program in Mild to Severe Parkinson's Disease: A Retrospective and Observational Study

**DOI:** 10.3389/fneur.2021.626041

**Published:** 2021-03-18

**Authors:** Mario Meloni, Francesca Lea Saibene, Sonia Di Tella, Monica Di Cesare, Francesca Borgnis, Raffaello Nemni, Francesca Baglio

**Affiliations:** ^1^IRCCS Fondazione Don Carlo Gnocchi ONLUS, Milan, Italy; ^2^Department of Psychology, Università Cattolica del Sacro Cuore, Milan, Italy; ^3^Università degli Studi di Milano, Milan, Italy

**Keywords:** parkinson's disease, neurorehabilitation, multidisciplinary program, cognitive therapy, physical therapy

## Abstract

Parkinson's disease (PD) is a neurodegenerative disorder characterized by motor (resting tremor, rigidity, bradykinesia, postural instability, and gait disturbances) and nonmotor symptoms (cognitive, neuropsychiatric, and autonomic problems). In recent years, several studies demonstrated that neurorehabilitation therapy is an effective treatment in addition to pharmacological personalized interventions in persons with PD (PwPD). The main aim of this study was to explore the short-term changes in functional, cognitive, and geriatric domains after a multidimensional rehabilitation program in PwPD (as primary condition) in mild–moderate (M-Ms) to severe (Ss) stages. Our second aim was to compare the effects of multidimensional rehabilitation in M-Ms versus Ss of PD. Twenty-four PwPD in M-Ms to Ss [age (mean ± SD) = 76.25 ± 9.42 years; male/female = 10/14; Hoehn and Yahr (median; IQR) = 4.00; 1.75] were included in a retrospective, observational study. Motor, cognitive, functional, and neuropsychiatric aspects were collected in admission (T0) and in discharge (T1). PwPD were involved in a person-tailored (to individual's needs), inpatient, intensive (5–7 days per week), multidisciplinary (combining cognitive, physical, occupational, and speech therapies), comprehensive, and rehabilitative program. According to Movement Disorders Society Unified Parkinson's Disease Rating Scale III cutoff, PwPD were classified in M-Ms or Ss (M-Ms ≤59; Ss >59); 87.50% of our sample reported significant reduction of functional disability at Barthel Index (*p* < 0.001). A significant improvement in Token test (*p* = 0.021), semantic fluency (*p* = 0.036), Rey's Figure-Copy (*p* < 0.001), and Raven's Colored Progressive Matrices (*p* = 0.004) was observed. The pain intensity perception (*p* < 0.001) and the risk of developing pressure ulcers (*p* < 0.001) as assessed, respectively, by the Numeric Rating Scale and by the Norton Scale were improved. With regard to the second aim, in M-Ms group, we found a positive correlation between the number of neuromotor sessions and the change in functional disability and language comprehension; in the Ss group, on the other hand, despite a higher number of hospitalization days, the total number of completed sessions was positively associated with the change in visuoconstructional abilities. Our findings suggest that an intensive, inpatient, and multidisciplinary rehabilitation program may improve functional abilities, some strategic cognitive functions, and geriatric aspects in PwPD with mild–moderate motor impairment.

## Introduction

Parkinson's disease (PD) is considered a multiple system neurodegenerative disorder with early prominent death of dopaminergic neurons in the substantia nigra pars compacta.

At time of diagnosis, the clinical hallmarks of the disease are represented by “the motor triad”: bradykinesia, rigidity, and resting tremor. Nowadays, it is known that in many persons with PD (PwPD), the neurodegenerative process produces a wide range of non-motor symptoms (such as cognitive, psychiatric, and autonomic symptoms), some of which precede the motor dysfunction by more than a decade (1). Psychiatric symptoms may be detected in *de novo* drug-naive patients, and they are not represented differently in the three main motor subtypes (akinetic-rigid, tremor-dominant, and mixed) (2).

To date, several drugs and surgical approaches are available for treating motor symptoms such as levodopa, dopamine agonists, monoamine oxidase B (MAO-B)/catechol-O-methyltransferase inhibitors, apomorphine infusion, levodopa–carbidopa intestinal gel infusion, and deep brain stimulation (DBS) (3).

However, the aforementioned strategies fail to prevent or delay the progressive neurodegenerative process, and patients continue to develop a progressive deterioration of their functional status with a loss of independence and a decline in quality of life (QoL). Increased disability is largely related to the increase of “axial” disturbances such as posture, balance, and gait, along with increased risk of falls. Moreover, even with optimal medical or surgical management, non-motor symptoms are suboptimally controlled. Therefore, the appearance over time of motor and non-motor complications induced by dopaminergic drugs (dyskinesias, motor fluctuations, dopamine dysregulation syndrome, impulse control disorders) results in detrimental impact on patients' clinical conditions and their QoL, emphasizing the need for non-pharmacological therapies (4).

In particular, cognitive impairment is related to QoL deterioration and functional disability in PwPD and imposes considerable burden on the caregiver (5).

To date, neurosurgical procedures and optimal pharmacological management have been shown ineffective in improving cognitive functions, even though MAO-B inhibitors may improve cognitive symptoms during wearing-off (6).

DBS-related cognitive outcomes are heterogeneous (7). A recent meta-analysis suggested that DBS results in decreased global cognition (8). Moreover, a progressive decrease in verbal fluency after DBS is consistently reported (9).

In recent years, several interventional studies have reported that the neurorehabilitation therapy is an effective treatment in addition to optimal medical therapy in PwPD (4, 10–17). Several studies are beginning to support the importance of intensity and complexity of physical activities to achieve benefits in motor function, cognition, and QoL in PwPD (18, 19). In particular, a recent phase 2 randomized clinical trial utilizing high-intensity treadmill exercise in individuals with new-onset PD found significantly less worsening of motor function in the high-intensity exercise group compared to the usual care group (20).

Interestingly, action observation therapy has been demonstrated effective in improving cognitive abilities of PwPD if it is used within a dual task framework (21).

To date, there are still few studies in which the rehabilitation program is conducted in an inpatient setting with intensive and multidimensional approach. The global impact of the rehabilitation programs may be greater if implemented in an inpatient setting. In the inpatient environment, PwPD can receive adequate management for several complex motor and non-motor symptoms. An inpatient multidisciplinary rehabilitation program ensures daily therapies in an environment that allows for regular monitoring of functional status and medication response, as well as an integrated and intense tailored physical rehabilitation (22).

Unfortunately, PwPD as the primary diagnosis are not typically referred for inpatient rehabilitation. Indeed, PwPD are often admitted with the aim of receiving inpatient hospital services following a cardiopulmonary, orthopedic, or general medical procedure or more rarely to improve the management of dopaminergic therapy.

With this background, the main aim of this study was to explore the short-term changes in functional, cognitive, and geriatric domains of an intensive, inpatient, multidisciplinary, and person-tailored rehabilitative program in PwPD (as primary condition) in mild–moderate (M-Ms) to severe (Ss) stages. Our second aim was to compare the effects of multidimensional rehabilitation in mild–moderate stage vs. severe stage of PD.

## Materials and Methods

### Study Design and Sample

This is a retrospective, observational study, in which 24 PwPD accessed the NeuroRehabilitation Unit of the S. Maria Nascente Center, IRCCS Fondazione Don Carlo Gnocchi ONLUS in Milan, were included.

The study was performed in accordance with the principles of the Helsinki Declaration and by previous approval from the IRCSS Fondazione Don Carlo Gnocchi ONLUS Ethics Committee.

All medical records and data extracted and included in the present study concerned patients with the following characteristics: [1] diagnosis of PD according to the United Kingdom Parkinson's Disease Society Brain Bank (23); [2] involvement in an inpatient personalized and multidimensional rehabilitation program during the period of hospitalization in our NeuroRehabilitation Unit; [3] the presence of an initial evaluation (T0 = within 1 week of admission) and a final assessment (T1 = the last days before discharge) regarding the rehabilitation treatment carried out; in particular, in admission and in discharge, they had to be present and filled in: a functional evaluation (performed by a physiatrist), a cognitive assessment (performed by a neuropsychologist), and an evaluation of common geriatric aspects (performed by a neurologist); [4] signature of a written informed consent that allows the use of clinical–medical data for research purposes.

Every professional who conducted rehabilitation sessions (physiotherapist, neuropsychologist, speech therapist, and occupational therapist) recorded the number of sessions given to each PwPD and its contents. A database containing all the clinical information from the patient's medical records was created; all pharmacological treatment was extrapolated, and the levodopa equivalent daily dose (LEDD) was calculated for each patient (24).

### Information and Variables Collected

The main data and variables extracted from the medical records were [1] the Barthel Index (BI) (total score and single item score) as a measure of PwPD's functional disability in daily life (25); [2] the Mini-Mental State Examination (MMSE) (26, 27), Token test (28), Phonemic and Semantic Fluency (29), Copy and Recall Rey's Figure (30), Raven's Colored Progressive Matrices (31) as measures of PwPD's cognitive functioning; and (3) measures of common geriatric aspects: (a) Numeric Rating Scale (NRS) (32) to detect the presence and severity of pain, (b) Norton Scale (33) to take over the risk of contracting pressure ulcers, and (c) Conley Scale (34) to assess the fall risk.

The Movement Disorders Society Unified Parkinson's Disease Rating Scale Part III-Motor Examination (MDS-UPDRS) Part III (35) was filled out at baseline.

### Intervention

All PwPD were involved in an intensive, tailored to individual's needs, specialized, inpatient, goal-based multidisciplinary rehabilitation program.

The primary aim of the rehabilitation program was to increase and promote functional, motor, and cognitive abilities, as well as to optimize the subjects' medication regimens.

In order to achieve this aim, all PwPD were involved in a combination of physical, cognitive, occupational, and speech therapy for a minimum of two daily rehabilitative sessions for 5–7 days per week (Table 1).

**Table 1:** Summary table of the interventions and rehabilitation sessions carried out during the period of hospitalization on our PwPD sample.

	**Physical therapy**	**Cognitive therapy**	**Occupational therapy**	**Speech therapy**
% of PwPD involved in each therapy	100,00	79,17	100,00	75,00
No. of therapy sessions [means (±SD)], independently of the hospitalization days	32,26 (±11.76)	16,45 (±11.14)	13,96 (±6.79)	10,13 (±8.83)

All admitted patients were enrolled in an intensive rehabilitation program consisting of daily sessions from Monday to Sunday for a total of at least 500 min a week.

The program was based on a multidisciplinary evaluation assessing the patients' needs and the possible goals, performed by a neurologist, together with a physical medicine and rehabilitation specialist. Interventions may include physical therapy (stretching, postural changes, gait exercises, balance, and postural control), occupational therapy (functional and goal-based exercises in order to readapting the use of daily tools and performing everyday tasks to recover personal autonomy and to improve targeted domains of QoL), cognitive rehabilitation (paper-and-pencil and computerized activities), and speech and swallowing rehabilitation (exercises to improve speech prosody and articulation, meal monitoring, and learning strategies for a proper ingestion of liquids and foods). The duration of the admission was established following an intermediate reassessment of the program and goals performed by the multidisciplinary rehabilitation team (physiotherapist, neuropsychologist, speech therapist, and occupational therapist) after 2–3 weeks of admission.

### Statistical Analyses

Statistical analyses were performed using SPSS 24.0 and jamovi (version 1.2) software (https://www.jamovi.org). Parametric and non-parametric statistics were adopted as appropriate. The PwPD sample was characterized in terms of age, sex, and clinical variables at the baseline.

For the MDS-UPDRS Part III, in addition to the total score, seven factor scores were calculated: factor 1: midline function; factor 2: rest tremor; factor 3: rigidity; factor 4: bradykinesia right upper extremity; factor 5: bradykinesia left upper extremity; factor 6: postural and kinetic tremors; and factor 7: lower limb bradykinesia (35).

For all outcome measures (domains of functional disability, cognitive functioning, and geriatric aspects), changes or variation scores were calculated from T1 to T0, obtaining Δ values. Paired-samples *t*-test was used to evaluate differences between baseline and after the multimodal rehabilitative intervention on LEDD. For the paired-samples *t*-test, effect size was given by Cohen *d*.

In order to account for relevant aspects related to delivery of treatment, PwPD's performance on outcome measures was analyzed using the jamovi module (34) (GAMLj: general analyses for linear models; retrieved from https://gamlj.github.io/) implemented in the software jamovi (version 1.2.25.0) software (https://www.jamovi.org). Generalized linear mixed models (GLMMs) were performed to evaluate score differences across the two time points (baseline and posttreatment).

Different test scores were used as dependent variables (one for each model), and time was considered as a fixed effect. To account for subject-specific variability, each subject was used as a random factor in all the models. The number of rehabilitative sessions, hospitalization days, and MMSE at the baseline were used as covariates as potential confounding variables. GLMMs final models were as follows: outcome measure ~1 + time + hospitalization days + number of rehabilitative sessions + MMSE at the baseline +(1 | subject), and only for MMSE, global cognitive outcome measure ~1 + time + hospitalization days + number of rehabilitative sessions (1 | subject).

A χ^2^ test was calculated to compare proportions between patients that remained stable after the intervention with them who reported an increase in the functional assessment evaluated by the BI.

Then, Spearman rank correlation coefficients (rS) were computed between changes or variation scores in all clinical outcomes and the seven UPDRS domains scores at the baseline.

Moreover, PwPD were classified in mild–moderate stage (M-Ms) or in severe stage (Ss) according to MDS-UPDRS Part III cutoff points (mild–moderate stage ≤59, severe stage >59) (36). The number of completed sessions was compared between the two groups.

Spearman rank correlation coefficients (rS) between the change scores (Δ) in clinical outcomes and the number of sessions were calculated in both the M-Ms and Ss groups. Spearman rank correlation coefficients (rS) between the change scores (Δ) in LEDD, neuropsychological measures, and BI were also computed. We interpreted the magnitude of correlation (effect size) as follows: 0.1–0.3 as a weak effect, 0.4–0.6 as a moderate effect, and 0.7 and higher as a strong effect (37).

All tests were 2-tailed, and *p*-values lower than 0.05 were considered statistically significant.

## Results

Sociodemographic and clinical characteristics as well as the measures of comorbidity and somatic health [Cumulative Illness Rating Scale (Severity Index and Comorbidity Index)] (38) of our sample are reported in Table 2.

**Table 2:** Demographical and clinical characteristic of PwPD sample at T0.

	**PwPD sample**	
	***n*** **=** **24**	
Males/females (*n*)	10/14	
Age (years), mean (SD)	76.25	9.42
Education (years), mean (SD)	8.79	3.88
Weight (kg), mean (SD)	67.35	15.14
Height (m), mean (SD)	1.64	0.11
BMI, mean (SD)	25.46	6.89
Hospitalization (days), mean (SD)	44.17	11.62
M-Ms (days), mean (SD)	37.90	10.90
Ss (days), mean (SD)	48.60	10.20
Disease duration (years), mean (SD)	10.43	6.15
H&Y, mean (SD)	3.88	0.91
M-Ms, mean (SD)	3.40	1.10
Ss, mean (SD)	4.21	0.58
Total score MDS-UPDRS Part III, mean (SD)	62.21	19.61
Factor 1: midline function, mean (SD)	21.29	6.90
Factor 2: rest tremor, mean (SD)	2.50	4.11
Factor 3: rigidity, mean (SD)	11.63	4.54
Factor 4: bradykinesia right upper extremity, mean (SD)	6.42	2.81
Factor 5: bradykinesia left upper extremity, mean (SD)	7.25	2.92
Factor 6: postural and kinetic tremors, mean (SD)	2.92	3.57
Factor 7: lower limb bradykinesia, mean (SD)	10.21	3.98
CIRS		
Severity Index, mean (SD)	1.40	0.16
Comorbidity Index, mean (SD)	2.00	1.07
Item 14: psychiatric-behavioral disorders (including dementia, depression, anxiety, agitation, psychosis); 1: absent; 2: mild; 3: moderate; 4: severe; 5: very serious, n (%) [*n* = 22 PwPD]	Distribution frequencies: 1: *n* = 4 (18.2%) 2: *n* = 9 (40.9%) 3: *n* = 9 (40.9%)	
LEDD, mean (SD)	785.58	541.04

*BMI, body mass index; H&Y, Hoehn & Yahr stages; MDS-UPDRS, Movement Disorders Society Unified Parkinson's Disease Rating Scale; CIRS, Cumulative Illness Rating Scale; LEDD, levodopa equivalent daily dose*.

Throughout their length of stay [hospitalization days (mean ± SD: 44.17 ± 11.62 days); total number of rehabilitation sessions (72.00 ± 28.61)], all PwPD were involved in a combination of physical therapy (mean number of sessions ± SD) (32.26 ± 11.76), cognitive therapy (16.45 ± 11.14); occupational therapy (13.96 ± 6.79), and speech therapy (10.13 ± 8.83) for a minimum of two sessions per day, 5–7 days per week.

### Prerehabilitation–Postrehabilitation Comparison

Results of GLMMs performed to evaluate score differences across the two time points (T0 and T1) are summarized in Table 3. In these models, the number of rehabilitative sessions, hospitalization days, and MMSE at the baseline were used as covariates in order to explore the impact of potential confounding variables.

**Table 3:** Comparison from baseline and post intervention assessments in our PwPD sample (*n* = 24).

	**Baseline**		**Postintervention**					
	**Mean**	**SD**	**Mean**	**SD**	**Time**	**Hospitalization days**	**No. of sessions**	**MMSE at baseline**
					***F*(df)**	***F*(df)**	***F*(df)**	***F*(df)**
					***p* value**	***p* value**	***p* value**	***p* value**
**Functional disability**								
BI total score	56.46	17.10	69.38	20.97	48.98 (1.21)	2.50 (1.18)	0.08 (1.18)	10.99 (1.18)
					<0.001	0.131	0.781	0.004
**Cognitive functioning**								
MMSE	24.79	5.82	25.68	5.06	3.25 (1.18)	0.56 (1.20)	0.01 (1.20)	-
					0.088	(0.463)	0.941	
Token test	27.50	6.10	29.13	5.02	6.64 (1,15)	0.18 (1,18)	1.33 (1,19)	46.84 (1,16)
					0.021	0.680	0.264	<0.001
Phonemic fluency	22.18	9.99	25.12	10.04	4.39 (1,18)	4.34 (1,20)	0.86 (1,21)	25.52 (1,17)
					0.051	0.050	0.365	<0.001
Semantic fluency	24.88	9.82	28.00	10.46	5.30 (1.16)	1.72 (1.18)	1.36 (1.19)	21.24 (1.19)
					0.036	0.205	0.257	<0.001
Copy Rey's figure	21.83	9.98	29.00	9.85	19.88 (1.12)	3.36 (1.17)	0.14 (1.16)	14.23 (1.14)
					<0.001	0.085	0.717	0.002
Recall Rey's figure	9.25	8.49	11.55	6.04	1.83 (1.11)	0.64 (1.15)	0.33 (1.15)	3.11 (1.13)
					0.202	0.435	0.577	0.102
Raven's colored progressive matrices	19.40	6.86	21.80	7.16	11.96 (1.14)	0.97 (1.18)	3.86 (1.18)	8.55 (1.18)
					0.004	0.337	0.065	0.009
**Geriatric aspects**								
Numeric rating scale of pain	5.72	3.49	2.61	2.62	17.62 (1.15)	3.83 (1.18)	0.00 (1.17)	0.77 (1.15)
					<0.001	0.066	0.990	0.395
Norton scale	14.61	1.99	16.74	2.28	31.03 (1.21)	0.80 (1.18)	0.43 (1.18)	4.63 (1.18)
					<0.001	0.382	0.520	0.045
Conley scale	4.21	2.04	3.53	1.71	3.84 (1.17)	3.63 (1.14)	2.96 (1.14)	0.03 (1.14)
					0.067	0.078	0.108	0.859

*BI, Barthel Index; df, degrees of freedom; MMSE, Mini-Mental State Examination*.

#### Functional Disability

Significant differences after the multimodal intervention emerged in the BI total score [*F*_(1, 21)_ = 48.98; *p* < 0.001]. Overall, comparison between proportions showed that 87.50% of patients reported higher scores vs. 12.50% of patients who remained stable in terms of the BI total score [$\chi_{\left(1\right)}^{2}$ = 13.50; *p* < 0.001]. MMSE at baseline had a significant impact on changes after treatment in BI total score [*F*_(1, 18)_ = 10.99; *p* = 0.004].

Interestingly, the change score in the functional disability (ΔBI total score) was negatively associated with the severity of motor symptoms, in more details with factor 4: bradykinesia right upper extremity (rS = −0.45; *p* = 0.028) and factor 5: bradykinesia left upper extremity (rS = −0.51; *p* = 0.012). Considering single item score of the BI, factor 4: bradykinesia right upper extremity also correlated with mobility change score (rS = −0.44; *p* = 0.033); factor 5: bradykinesia left upper extremity also correlated with dressing change score (rS = −0.45; *p* = 0.026); bladder change score (rS = −0.43; *p* = 0.038); and stairs change score (rS = −0.46; *p* = 0.025).

#### Cognitive Functioning

With respect to the neuropsychological evaluation, a significant improvement was observed in language [Token test: *F*_(1, 15)_ = 6.64; *p* = 0.021; semantic fluency: *F*_(1, 16)_ = 5.30; *p* = 0.036] in visuoconstructional abilities [Copy Rey's Figure: *F*_(1, 12)_ = 19.88; *p* < 0.001] and in abstract reasoning [Raven's Colored Progressive Matrices: *F*_(1, 14)_ = 11.96; *p* = 0.004]. MMSE at baseline had a significant impact on changes after treatment in Token test [*F*_(1, 16)_ = 46.84; *p* < 0.001]; phonemic fluency [*F*_(1, 17)_ = 25.52; *p* < 0.001]; semantic fluency [*F*_(1, 19)_ = 21.24; *p* < 0.001]; Copy Rey's Figure [*F*_(1, 14)_ = 14.23; *p* < 0.002]; and Raven's Colored Progressive Matrices [*F*_(1, 18)_ = 8.55; *p* < 0.009].

#### Geriatric Aspects

After the multidisciplinary rehabilitation program, significant differences were also reported for the NRS [*F*_(1, 15)_ = 17.62, *p* = 0.001] and for the Norton Scale [*F*_(1, 21)_ = 31.03, *p* < 0.001]. There was no statistically significant association between change scores in geriatric aspects and the number of rehabilitative sessions. MMSE at baseline had a significant impact on changes after treatment in the Norton Scale [*F*_(1, 18)_ = 4.63; *p* = 0.045].

Noteworthy, reduction in LEDD was observed comparing values between T0 and T1 (mean LEDD T0 = 785.58 ± 541.04; mean LEDD T1 = 695.75 ± 473.05), although not statistically significant (*p* = 0.111; Cohen's *d* = 0.339).

## M-MS VS. SS Group Analysis

Splitting our sample according to MDS-UPDRS Part III cutoff points, 10 patients were classified in the M-Ms group and 14 patients in the Ss group. In relation to the duration and intensity of the multimodal intervention, the total number of rehabilitative sessions (summing up all cognitive, neuromotor, and occupational therapy sessions) and the total number of hospitalization days was higher in the Ss group than in the M-Ms group [total sessions: *t*_(22)_ = 2.76; *p* = 0.011; neuromotor sessions: *t*_(22)_ = 2.59; *p* = 0.017; occupational therapy sessions: *t*_(22)_ = 2.37; *p* = 0.027; days of hospitalization: *t*_(22)_ = 2.47; *p* = 0.022].

In the M-Ms group, the number of neuromotor sessions was positively correlated with the change in functional disability [ΔBI total score (M-Ms group): rS = 0.66 (moderate effect); *p* = 0.038; (Ss group): rS = −0.07 (weak effect); *p* = 0.804] and in language comprehension [ΔToken test (M-Ms group): rS = 0.98 (strong effect); *p* = 0.005; (Ss group): rS = 0.55 (moderate effect); *p* = 0.077]. In the Ss group, however, the total number of completed sessions was positively associated with the change in visuoconstructional abilities [ΔCopy Rey's Figure (M-Ms group): rS = 0.11 (weak effect); *p* = 0.796; (Ss group): rS = 0.93 (strong effect); *p* < 0.001]. The change in visuoconstructional abilities was also positively correlated with the number of neuromotor sessions [ΔCopy Rey's Figure (M-Ms group): rS = 0.50 (moderate effect); *p* = 0.667; (Ss group): rS = 0.68 (moderate effect); *p* = 0.044] and with the number of occupational therapy sessions [ΔCopy Rey's Figure (M-Ms group): rS = 0.22 (weak effect); *p* = 0.604; (Ss group): rS = 0.68 (moderate effect); *p* = 0.042]. In this last group, we observed a trend for the correlation between the number of neuromotor sessions and abstract reasoning [ΔRaven's Colored Progressive Matrices: (M-Ms group): rS = 0.26 (weak effect); p = 0.742; (Ss group): rS = 0.60 (moderate effect); *p* = 0.050].

## Discussion

The main finding of our study is that an intensive, inpatient, multidisciplinary rehabilitation program leads to significant short-term changes in PwPD with mild–moderate motor impairment at baseline, in particular with respect to functional status and in some strategic cognitive domains. In PwPD with severe motor impairment at baseline, the more intensive rehabilitation program was associated only with significant short-term changes in visuoconstructive abilities.

Most importantly, the improvement in several activities of daily living such as dressing, bladder control, chair transfer, ambulation, and stair climbing was inversely related with the severity of motor dysfunctions at baseline evaluation, in particular with upper extremity bradykinesia.

Several factors determine the functional impairment in PwPD. In particular, rigidity and bradykinesia tend to most greatly affect a patient's ability to work and perform activities of daily living.

In particular, as regards the upper limb, the bradykinesia may cause issues in dexterous activities such as using kitchen or work tools. It may also contribute to reducing the coordination in activities such as dressing, toileting, and transferring.

Effectively, some activities of daily living evaluated by the BI such as dressing and chair transfer require an appropriate movement speed of the upper limbs and manual dexterity, and this could explain the best rehabilitation treatment response in PwPD with lower bradykinesia severity at baseline.

Interestingly, during the rehabilitative hospitalization, the improvement in functional disability has made possible the reduction of the daily dose of dopaminergic medications, albeit not statistically significant. Indeed, it has been demonstrated that intensive rehabilitation, improving motor performances, and autonomy in activities of daily life, reduces the need to increase dopaminergic therapy (15, 39).

In the present study, PwPD showed significant improvements in language comprehension, visuoconstructional abilities, and abstract reasoning following cognitive therapy sessions.

Interestingly, we observed a significant effect of baseline global cognitive functions on the reported changes on outcome measures, implying that the treatment, regardless of the intensity, is effective only according to cognitive functioning at baseline.

Indeed, patients with lower global cognitive impairment are likely to maintain information about goals to be achieved over time, such as rehabilitation goals (40).

Our findings confirmed the beneficial effects of an intensive cognitive rehabilitation program in combination with multiple integrated interventions (physical therapy, occupational therapy, and speech therapy). Consistent with previous studies, we suppose that the benefits of cognitive treatments have been enhanced by combining cognitive remediation with motor function training and occupational interventions (41).

To date, few studies have investigated the neurobiological and neurofunctional modifications in PwPD after an integrative cognitive rehabilitation program (42–44). These studies support the idea of structural and functional brain changes related to the cognitive improvements after rehabilitation despite the neurodegenerative process.

In light of our results, it is arguable that the improvement in PwPD's cognitive performances (visuoperceptual and visuoconstructional abilities, language, and visual and verbal memory) may result in a significant reduction of disability as indicated by clear BI modification.

Additionally, improvement in functional performance driven by high-intensity rehabilitation program was associated with significant improvements in important geriatric aspects such as pain intensity perception, and the risk of developing pressure ulcers as assessed, respectively, by the NRS and by the Norton Scale.

Our findings strengthen the evidence about the efficacy of an inpatient multidisciplinary rehabilitation treatment on PwPD (15, 39, 45).

A still debated issue is whether a single or a multidisciplinary and integrated approach (speech, cognitive, occupational, and physical therapy) should be proposed for PwPD. Indeed, an excessively multifocused set might be affected by a low intensity of training.

In a recent study, a remarkable effect (persisting at 1-year follow-up) on motor symptom severity and QoL was demonstrated combining high intensity in an inpatient setting with a multidisciplinary approach. The multifaceted needs of PwPD vary considerably across disease stages so that different management approaches and interventions tailored to the individual conditions of patients are mandatory (16).

Despite compelling scientific evidence, an inpatient rehabilitation admission is typically not considered for patients with a progressive disease such as PD. An inpatient rehabilitation program can ensure many repetitions of the same task with consequent reinforcement of the beneficial effects. Moreover, it has been amply demonstrated that PwPD may benefit more from blocked practice than from random practice of exercise and physical therapy (46). Indeed, it is still debated if PwPD retain sufficient capacity to reacquire or to learn new skills. Typically, because of striatum dysfunction, the consolidation and automatization of learned motor and cognitive exercises are significantly affected in PwPD (47).

One of the major future challenges will be the ability to identify PwPD most likely to benefit from inpatient rehabilitation. In particular, it is important to identify at which level of motor impairment the multidisciplinary inpatient rehabilitation program may be of benefit. An important finding of our study is the evidence of beneficial effects of an intensive rehabilitation program in mild–moderate severity of motor symptoms. Indeed, despite the fewer number of rehabilitative sessions and hospitalization days, PwPD in M-Ms group showed the best improvements in degree of disability according to BI. Despite this, the positive association between the number of neuromotor, occupational, and speech therapy sessions and the improvements in the executive-constructional task performances observed in Ss of PwPD suggests that rehabilitation provides benefit in advanced motor impairment as well. However, in our study, the total numbers of rehabilitative sessions and hospitalization days were higher in the Ss group than in the M-Ms group. Indeed, subjects with a more advanced disease needed longer treatments without having the same benefit.

In light of our results, we speculate that an intensive inpatient multidisciplinary rehabilitation should be considered in early/moderate PD stages in order to optimize functions and slow progression of disability and to avoid longer hospitalization/rehospitalization and consequently higher financial costs.

Given the paucity of studies about the effectiveness of a cognitive, motor, and functional rehabilitation program, we claim that the present article is of great interest. Compared to many of the previous studies, our work examined the effect of an inpatient multidisciplinary rehabilitation treatment on PwPD with different disease severity (from mild to severe). Moreover, we investigated the effectiveness of an inpatient rehabilitation program on PwPD as the primary diagnosis instead of PwPD hospitalized as a result of cardiopulmonary, orthopedic procedure or to manage the dopaminergic therapy.

The main strength of the present investigation is the fair number of treated patients, although several methodological issues severely limit generalization of findings beyond the current sample.

The lack of a control group limited our ability to attribute the improvements observed specifically to rehabilitation or to the combination of pharmacological and rehabilitation interventions. In addition, improvements attributable to other aspects of an inpatient hospital admission cannot be ruled out. Moreover, this study did not include a short- and long-term follow-up after the discharge. As such, we were unable to verify the maintenance of cognitive and functional improvements over time. Additionally, we did not verify the effectiveness of intensive rehabilitation program on motor functions. Indeed, the MDS-UPDRS Part III was assessed only at baseline.

Although we are aware that this is a retrospective study in which we collected data and information from medical records, it is necessary to highlight some further limitations about the assessment phase on admission and discharge: in fact, no important data have been collected in the cognitive field (lacking data concerning, for example, executive functions) and in the neuropsychiatric field (there is no scale for detecting psychiatric disorders).

The interest to promote inpatient rehabilitation programs in PwPD as a means for eliciting improvement in functional status and cognitive performances has underscored the importance of identifying the tailored multidisciplinary approach in accordance to the disease stage and individual conditions of patients.

Our results suggest a potential role of inpatient multidisciplinary rehabilitation in driving activity-dependent functional improvement in mild–moderate PwPD's motor skills.

Our data showed that treatment intensity is not a mediating factor in determining posttreatment changes and that only PwPD with mild–moderate motor impairment can improve after a multidimensional treatment. However, it is important to clarify that the potential effect of multidimensionality was not explored.

Moreover, the longitudinal maintenance of changes should be examined to consider the periodicity with which PwPD should attend multiple integrated interventions.

Future tailored protocols aiming to integrate multimodal rehabilitation programs should also identify optimal parameters of frequency, intensity, duration, and task-specific exercises to improve effectiveness and tolerability.

More research is needed to evaluate the long-term effects on the functional outcome with longer periods of time and the possible benefits of booster sessions.

## Data Availability Statement

The raw data supporting the conclusions of this article will be made available by the authors, without undue reservation.

## Ethics Statement

The studies involving human participants were reviewed and approved by IRCSS Fondazione Don Carlo Gnocchi ONLUS Ethics Committee. The patients/participants provided their written informed consent to participate in this study.

## Author Contributions

MM, FLS, RN, and FBa: study concept, methodology, and design. FLS, MDC, and FBo: data selection, collection, and data-entry. SDT: methodology and statistical analyses. MM, FLS, and SDT: data interpretation and drafting the first version of the manuscript. RN and FBa: supervision and project administration. MM, FLS, SDT, MDC, FBo, RN, and FBa: critical review and revision of the manuscript. All the authors made changes and approved the final paper.

## Conflict of Interest

The authors declare that the research was conducted in the absence of any commercial or financial relationships that could be construed as a potential conflict of interest.
